# Identification of copper-related biomarkers and potential molecule mechanism in diabetic nephropathy

**DOI:** 10.3389/fendo.2022.978601

**Published:** 2022-10-18

**Authors:** Jie Ming, Si Ri Gu Leng Sana, Xijin Deng

**Affiliations:** ^1^ Department of Urology, The First Affiliated Hospital of Harbin Medical University, Harbin, China; ^2^ Department of Anaesthesiology, The First Affiliated Hospital of Harbin Medical University, Harbin, China; ^3^ Department of Anaesthesiology, The Second Affiliated Hospital of Harbin Medical University, Harbin, China

**Keywords:** diabetic nephropathy, copper-related genes, WGCNA, transcriptional factors, miRNAs, immune cell infiltration

## Abstract

**Background:**

Diabetic nephropathy (DN) is a chronic microvascular complication in patients with diabetes mellitus, which is the leading cause of end-stage renal disease. However, the role of copper-related genes (CRGs) in DN development remains unclear.

**Materials and methods:**

CRGs were acquired from the GeneCards and NCBI databases. Based on the GSE96804 and GSE111154 datasets from the GEO repository, we identified hub CRGs for DN progression by taking the intersection of differentially expressed CRGs (DECRGs) and genes in the key module from Weighted Gene Co-expression Network Analysis. The Maximal Clique Centrality algorithm was used to identify the key CRGs from hub CRGs. Transcriptional factors (TFs) and microRNAs (miRNAs) targeting hub CRGs were acquired from publicly available databases. The CIBERSORT algorithm was used to perform comparative immune cell infiltration analysis between normal and DN samples.

**Results:**

Eighty-two DECRGs were identified between normal and DN samples, as were 10 hub CRGs, namely *PTGS2, DUSP1, JUN, FOS, S100A8, S100A12, NAIP, CLEC4E, CXCR1*, and *CXCR2.* Thirty-nine TFs and 165 miRNAs potentially targeted these 10 hub CRGs. *PTGS2* was identified as the key CRG and *FOS* as the most significant gene among all of DECRGs. *RELA* was identified as the hub TF interacting with *PTGS2* by taking the intersection of potential TFs from the ChEA and JASPAR public databases. let-7b-5p was identified as the hub miRNA targeting *PTGS2* by taking the intersection of miRNAs from the miRwalk, RNA22, RNAInter, TargetMiner, miRTarBase, and ENCORI databases. Similarly, *CREB1*, *E2F1*, and *RELA* were revealed as hub TFs for *FOS*, and miR-338-3p as the hub miRNA. Finally, compared with those in healthy samples, there are more infiltrating memory B cells, M1 macrophages, M2 macrophages, and resting mast cells and fewer infiltrating activated mast cells and neutrophils in DN samples (all *p<* 0.05).

**Conclusion:**

The 10 identified hub copper-related genes provide insight into the mechanisms of DN development. It is beneficial to examine and understand the interaction between hub CRGs and potential regulatory molecules in DN. This knowledge may provide a novel theoretical foundation for the development of diagnostic biomarkers and copper-related therapy targets in DN.

## Introduction

Diabetes mellitus (DM) currently affects more than 350 million individuals worldwide. The number of affected is expected to exceed 600 million by 2045 (1, 2). Diabetic nephropathy (DN) is a chronic microvascular complication that develops in approximately 30% of all patients with DM. DN is characterized by a persistent increase of albuminuria, defined as >300 mg in a 24-h period or an albumin-to-creatinine ratio >300 mg/g, decreased glomerular filtration rate (GFR), increased blood pressure, and excessive risk of cardiovascular complications and concomitant diabetic retinopathy (3). Despite multifactorial treatments that include glycemic, lipid, and blood pressure control (4), renin-angiotensin system inhibitors including angiotensin-converting enzyme inhibitors (5) and angiotensin receptor blockers (6), peroxisome proliferator-activated receptor agonists (7), PKC antagonist (8), the angiotensin-converting enzyme inhibitor pyridoxamine (9), and endothelin receptor inhibitors like atrasentan (10), DN is the main cause of end-stage renal disease (ESRD) with a high disability and mortality in western countries. Therefore, it makes sense to consider new therapeutic approaches for DN based on novel potential mechanisms.

Copper (Cu) is a crucial trace element that is a structural component of enzymes. Cu regulates signaling pathways with many key roles in various biological processes, such as antioxidant activity, mitochondrial respiration, metabolic reprograming, enhanced proliferation (cuproplasia), and angiogenesis (11). Normally, Cu concentrations in cells are kept at an extremely low level by active homeostatic mechanisms that function across concentration gradients (12, 13). The elevated intracellular concentration of Cu exceeding a threshold can be cytotoxic and result in cell death. However, the specific and complete mechanisms of Cu-induced cell death remain unclear and contradictory despite decades of research. Some research indicates that the massive accumulation of reactive oxidative species (ROS) caused by increased level of Cu ions in cells results in apoptosis or autophagy (14–19). However, other studies indicate that Cu-induced toxicity is dependent on the inhibition of the ubiquitin-proteasome system (20–23). A recent study by Tsvetkov et al. provided the novel proposal that Cu targets lipoylated tricarboxylic acid cycle proteins to induce cell death (cuproptosis) independent of the currently known forms of cell death, including ferroptosis, apoptosis, necroptosis, and oxidative stress (24). Seven key genes are involved in cuproptosis. These include Ferredoxin 1 encoded by *FDX1*. FDX1 is a reductase responsible for the reduction of Cu^2+^ to Cu^1+^ and is an upstream regulator of protein lipoylation. The remaining six genes include three genes that encode proteins of the lipoic acid pathway [i.e., lipoyl synthase (*LIAS*), lipolytransferase 1 (*LIPT1*), and dihydrolipoamide dehydrogenase (*DLD*)] and three genes that synthesize protein targets of lipoylation [pyruvate dehydrogenase E1 subunit alpha 1 (*PDHA1*), pyruvate dehydrogenase E1 subunit beta (*PDHB*), and dihydrolipoamide S-acetyltransferase (*DLAT*)]. It is believed that excessive cellular Cu results in aggregation of lipoylated proteins and degradation of Fe-S cluster proteins, leading to proteotoxic stress and subsequently cuproptosis (24).

Some Cu agents have shown promising potential in the treatment of various diseases. For example, Skrott et al. tested the cytotoxicity of the ditiocarb-copper complex (CuET) on a panel of Velcade/Carfilzomib-adapted human cell lines. CuET was implicated as a promising therapeutic agent for patients with recurrent, Velcade-resistant multiple myeloma (20). A statistically significant doubling of progression-free survival time was observed with elesclomol plus paclitaxel compared to that of paclitaxel alone in a phase III clinical trial for melanoma (25). Allensworth et al. found that disulfiram produced oxidative stress-mediated apoptosis involving the inhibition of NF-κB signaling and the reduction of levels of aldehyde dehydrogenase and antioxidant in a multi-inflammatory breast cancer cellular model (26). However, few studies have focused on the role of Cu agents in DN (27).

In the present study, we aimed to identify hub Cu-related genes (CRGs) and predict the potential molecular regulation network. The findings may provide a novel theoretical foundation for the development of diagnostic biomarkers and therapy targets in DN.

## Materials and methods

### Datasets and pre-processing

Two DN-related microarray datasets (GSE96804 and GSE111154) were downloaded from the Gene Expression Omnibus (GEO) repository (https://www.ncbi.nlm.nih.gov/geo/). Both datasets were acquired from the GPL17586 platform.

R software (version 4.1.2) was used to perform data processing. The probes were mapped to the gene *via* Strawberry Perl (version 5.32.1.1) while no-load probes were removed. The mean value was calculated if more than one probes matched to the same gene. Normalization is a data analysis method that adjusts global properties of measurements for individual samples. This method helps to reduce the global differences of the data distribution (28). In our study, gene expression values were first converted to log_2_-transformed quantile-normalized signal intensity. Then the log_2_-transformed matrix was normalized *via* the *normalizeBetweenArrays* function of limma package to achieve consistency between arrays (29). Batch effects are subgroups of measures that exhibit qualitatively different behavior under different conditions. Batch effects can pose serious concerns about the validity of biological findings despite being unrelated to biological variables in medical research (28). Therefore, the *ComBat* function of SVA package was used to eliminate the batch effect after the normalization of gene expression values (30). Using the code “data=data[apply(data,1,sd)>0.5]”, we deleted less variable genes between normal and DN samples (standard deviation > 0.5). Ultimately, 3044 genes were subjected to Weighted Gene Co-expression Network Analysis (WGCNA).

### Use of WGCNA to identify gene modules most related to the development of DN

WGCNA is a systematic approach that can investigate the association between gene networks and a quantitative measure (referred to as sample trait). The analysis identifies co-expressed gene modules with pronounced biological significance (31). In our study, the binary indicator variable (DN status) was taken as the sample trait, which helped to find the co-expressed gene modules with DN. The WGCNA package was used to perform co-expression network analysis. We normalized the samples and removed the outlined samples to ensure the reliability of the network construction. Subsequently, the adjacent matrix that described the correlation strength between genes was obtained through the formula listed as follows:


$$a_{ij}=\;{\left|S_{ij}\right|}^{\beta }=\;\left|power\;\left[S_{ij},\;\beta \right]\right|$$

where i and j are two individual genes and S_ij_ represents the Pearson’s correlation coefficient between genes i and j. *Power* is a function where S_ij_ is the base and β is the power that is identified by *pickSoftThreshold* function. Subsequently, the adjacent matrix was transformed into a topological overlap matrix (TOM). We set the deepSplit value and minimum number of genes in the module as 2 and 50, respectively. Average linkage hierarchical clustering was performed to identify modules of closely interacting genes. A height cutoff of 0.25 was selected as the criterion to merge modules with similar gene profiles using the DynamicTreeCut algorithm.

To identify the biologically significant gene module, several metrics were included as the assessment criteria. These included module eigengenes (MEs), gene significance (GS), and module significance (MS) (31). MEs are defined as the first principal component of each gene module, which is regarded as a representative of all genes in a given module. The significant gene module is determined by the correlation coefficient between MEs and the corresponding disease status. GS is defined as the correlation between gene expression profiles and an external clinical trait including disease status (GS = -lg *p*). This metric helps to incorporate external clinical information into the co-expression network. The biological significance of the gene increases with the absolute value of GS. MS is defined as the average absolute GS measure for all genes within a given module. MS is used to screen and incorporate the significant gene module.

### Identification of hub CRGs

By searching the key word “copper” in GeneCards (https://www.genecards.org/) and National Center for Biotechnology Information (NCBI, https://www.ncbi.nlm.nih.gov/) database (only for genes of homo sapiens), we obtained 2142 CRGs summarized in **Supplementary Table 1**.

We first acquired differentially expressed CRGs (DECRGs) between normal and DN samples using the LIMMA package with the criteria of |log_2_ fold change| ≥ 1.0 and adjusted *p*< 0.05. The intersection of DECRGs and genes in the most biologically significant module identified by WGCNA were regarded as the hub CRGs. Venn diagram was plotted through the VennDiagram package.

### Prediction of transcription factors and microRNAs associated with hub CRGs

To explore the potential changes and molecular regulatory mechanisms happening at the transcriptional level for hub CRGs, we tried to decode the regulatory TFs and miRNAs using a network-based approach. Topologically credible TFs were identified from the JASPAR and ChEA repositories on the NetworkAnalyst platform (https://www.networkanalyst.ca/). The intersection of TFs from both repositories was used to obtain the hub TFs. Similarly, miRNAs targeting hub CRGs from six repositories, namely ENCORI (https://starbase.sysu.edu.cn/), miRWalk (http://mirwalk.umm.uni-heidelberg.de/), RNA22 (https://cm.jefferson.edu/rna22/), RNAInter (http://www.rnainter.org/), TargetMiner (https://www.isical.ac.in/~bioinfo_miu/targetminer20.htm), and miRTarBase (https://www.networkanalyst.ca/), were identified. The intersecting miRNAs were also identified and retained. Among these repositories, miRTarBase is the major experimentally validated database for miRNA-target RNA interactions (32).

### Functional enrichment analysis and protein–protein interaction network construction

The functional enrichment analysis was performed to identify the corresponding biological pathways involved in the hub CRGs. Gene ontology (GO) enrichment analysis interpreted the biological significance of genes from three perspectives: biological process (BP), cellular component (CC), and molecular function (MF). Kyoto Encyclopedia of Genes and Genomes (KEGG) pathway analysis was used to systematically assess gene function. The “Clusterprofiler” package was employed to automate the enrichment analysis of gene clusters. The “org.Hs.eg.db” package was used for conversion between gene IDs (33).

Proteins execute their respective important biological functions through mutual affinity within the cell to form the PPI network. The STRING database permits wide coverage and completeness of evidence sources by integrating all known and predicted physical and functional associations between proteins. Accordingly, this database has been chosen as one of the *European Core Data Resources* by the ELIXIR consortium (34) and is currently used by approximately 5000 users each day. The database collects and scores evidence of PPI from four sources: automated text mining of the literature, databases of annotated complexes or pathways and interaction experiments, predictions of computational interactions based on co-expression and conserved genomic context, and transfer of interaction evidence between organisms in a systematic manner (35). Data integration across various evidence sources improves the overall network quality (36–39). STRING was reported to have the greatest ability to recover a diverse collection of disease-associated gene sets among 21 human gene–gene interaction network databases (40). Accordingly, we created the PPI network to acquire the insights into cellular machinery operations through the STRING database (https://cn.string-db.org/). We set the minimum required interaction scores as the highest confidence (0.900) and hid the disconnected nodes to generate the PPI network. Cytoscape (version 3.9.0) software was used to further present and integrate biomolecular interaction networks. Subsequently, we used cytohubba (http://apps.cytoscape.org/apps/cytohubba) to rank and screen the central or targeted elements of the network. Cytohubba is a Cytoscape plugin comprising 11 methods for investigating networks from different viewpoints. The methods include Maximal Clique Centrality (MCC), Maximum Neighborhood Component, Density of Maximum Neighborhood Component, Closeness (Clo), EcCentricity (EC), Radiality (Rad), BottleNeck (BN), Stress (Str), Betweenness (BC), Edge Percolated Component, and Degree. Among these methods, we chose MCC, since it has been identified as the best method to screen hub CRGs (41).

### CIBERSORT immune cell infiltration analysis

Cell-type Identification By Estimating Relative Subsets Of RNA Transcripts (CIBERSORT) is a gene expression profile-based method for determining the cell composition of complex tissues. The method includes expression data of 22 immune cells (LM22) and performs better than other methods considering noise, unknown mixture components, and cell types (42). We used the CIBERSORT algorithm to analyze the normalized RNA sequence (RNA seq) data and acquire the immune infiltration status for each sample.

Pearson correlation coefficient was calculated to measure the correlation between the expression of hub gene and immune cell infiltration levels.

## Results

**Figure 1:**
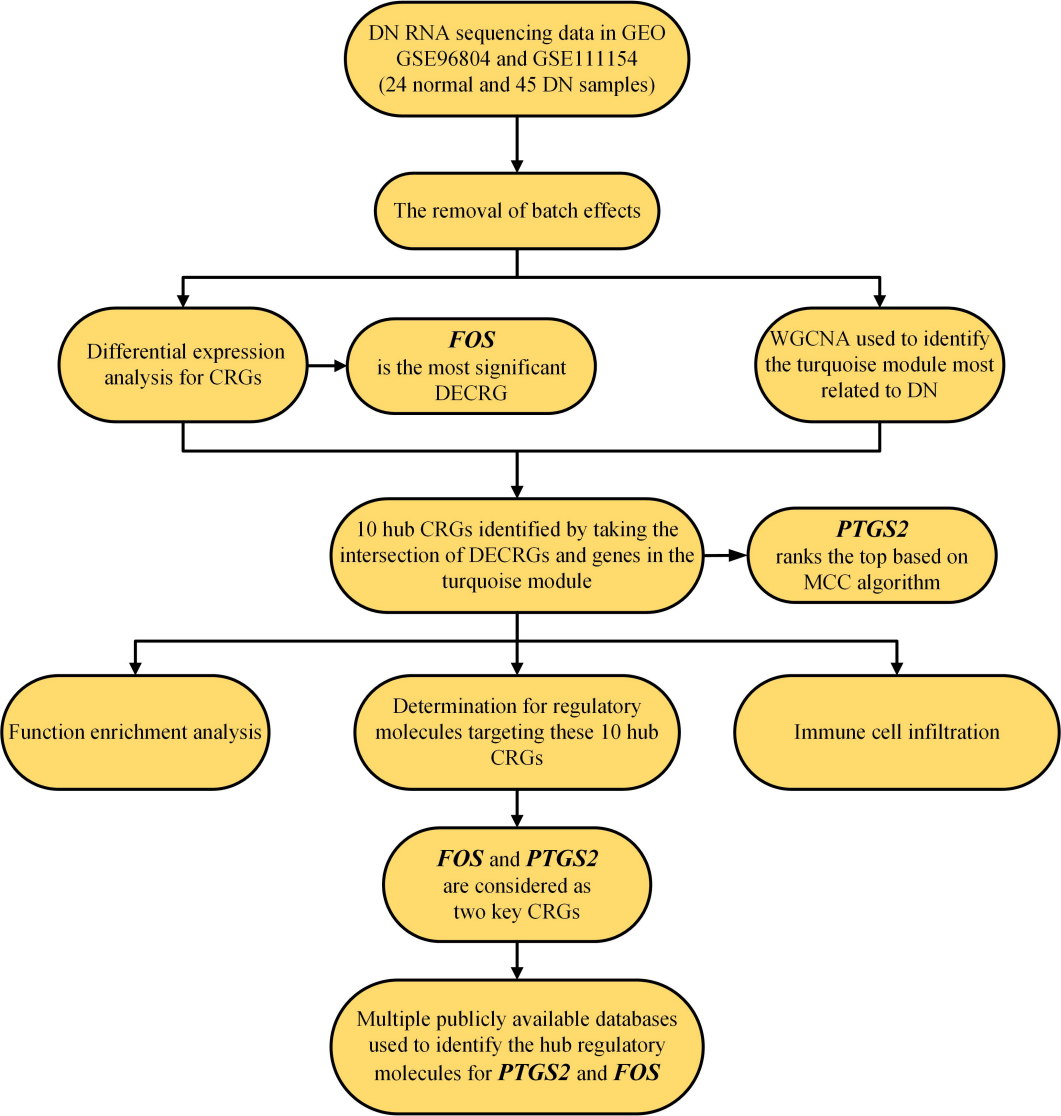
Flow chart of this study. CRG, copper-related gene; DECRGs, differentially expressed copper-related genes; and DN, diabetic nephropathy.

The flow chart of this study is shown in **Figure 1**.

### Removal of batch effects

**Figure 2:**
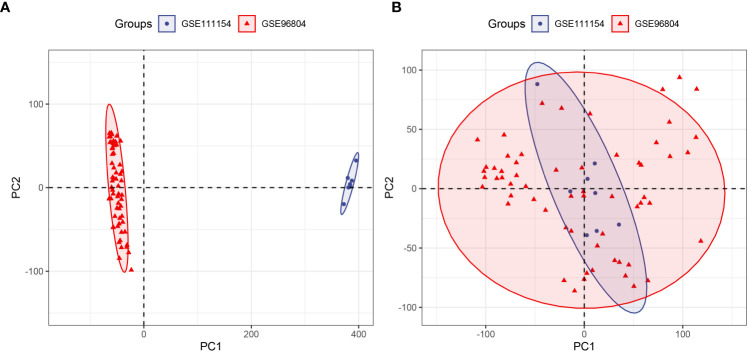
Principal component analysis (PCA) of two datasets (GSE96804 and GSE111154). The points of the scatter plot represent the samples without **(A)** and with **(B)** the removal of batch effects according to the first two principal components of gene expression profiles.

Despite the use of two datasets (GSE968034 and GSE111154) acquired from the same GPL17586 platform, there were still batch effects for the measurements affected by laboratory conditions, reagent lots, processing date, personnel differences, and other causes (28). Therefore, the *ComBat* function of the SVA package was used to eliminate the potentially unwanted sources of variation. Before the removal of batch effects, samples were clustered by batches based on principal component analysis (PCA) of unnormalized gene expression values (**Figure 2A**). By contrast, samples were all mixed together after removing batch effects based on the first two principal components (**Figure 2B**). The results confirmed the success of the removal of batch effects.

### DECRGs in DN

**Figure 3:**
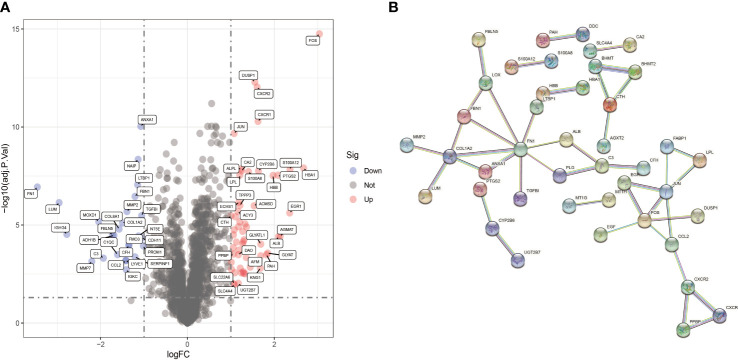
Identification of DECRGs. **(A)** Volcano plot of DECRGs between normal and DN samples. **(B)** PPI network for DECRGs in DN (80 nodes and 42 edges).

The GSE96804 and GSE111154 datasets in the GEO database were examined. A total of 69 samples were obtained, which included 24 normal samples and 45 DN samples. Eighty-two DECRGs were identified between normal and DN samples. Of these, 26 genes were up-regulated and 56 down-regulated (**Figure 3A**). The PPI network for DECRGs in DN was constructed with a minimum required interaction score of 0.900 and disconnected nodes hidden in the network, which included 80 nodes and 42 edges (**Figure 3B**). Notably, the PPI network revealed a close relationship between prostaglandin-endoperoxide synthase (PTGS2) and cytochrome P450 (CYP) family 2 subfamily B member 6 (CYP2B6).

### Analysis of module closely related to DN

**Figure 4:**
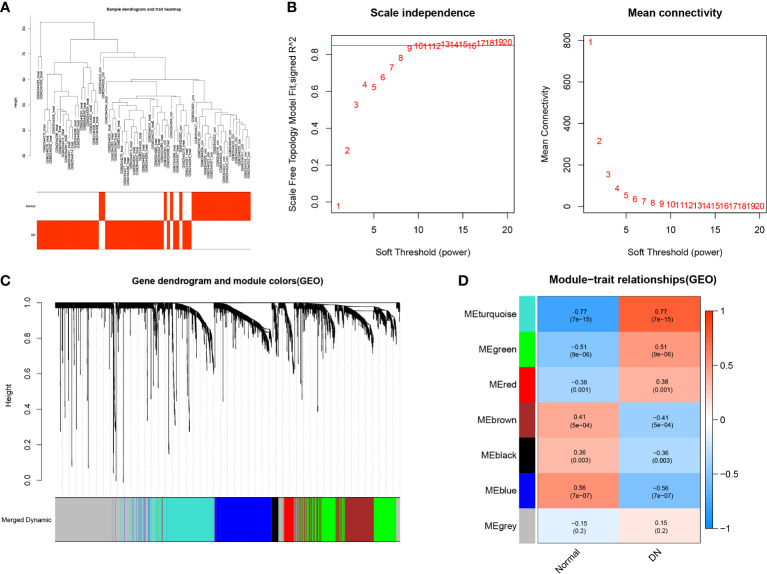
Weighted Gene Co-expression Network Analysis. **(A)** Clustering dendrogram of 69 samples. **(B)** Analysis of the scale free fit index for different soft-thresholding powers (β), and analysis of the mean connectivity for different soft-thresholding powers. **(C)** Dendrogram of differentially expressed genes clustered based on a dissimilarity measure (1-TOM). **(D)** Heatmap of correlations between different modules and clinical traits, including normal tissues and DN tissues.

The samples of the GSE96804 and GSE111154 datasets were clustered using the average linkage and Pearson’s correlation methods (**Figure 4A**). A β value of 13 was selected as the soft-thresholding power to ensure a scale free network (scale free *R* ^2^ = 0.856), as shown in **Figure 4B**. A total of seven gene modules were acquired *via* the average linkage hierarchical clustering (**Figure 4C**). The correlation between modules and DN status is depicted in **Figure 4D**. The turquoise module that includes 564 genes was most significantly associated with DN. This module was chosen for the further identification of hub CRGs (R =0.77, *p =*7e-15).

### Identification and analysis of 10 hub CRGs

**Figure 5:**
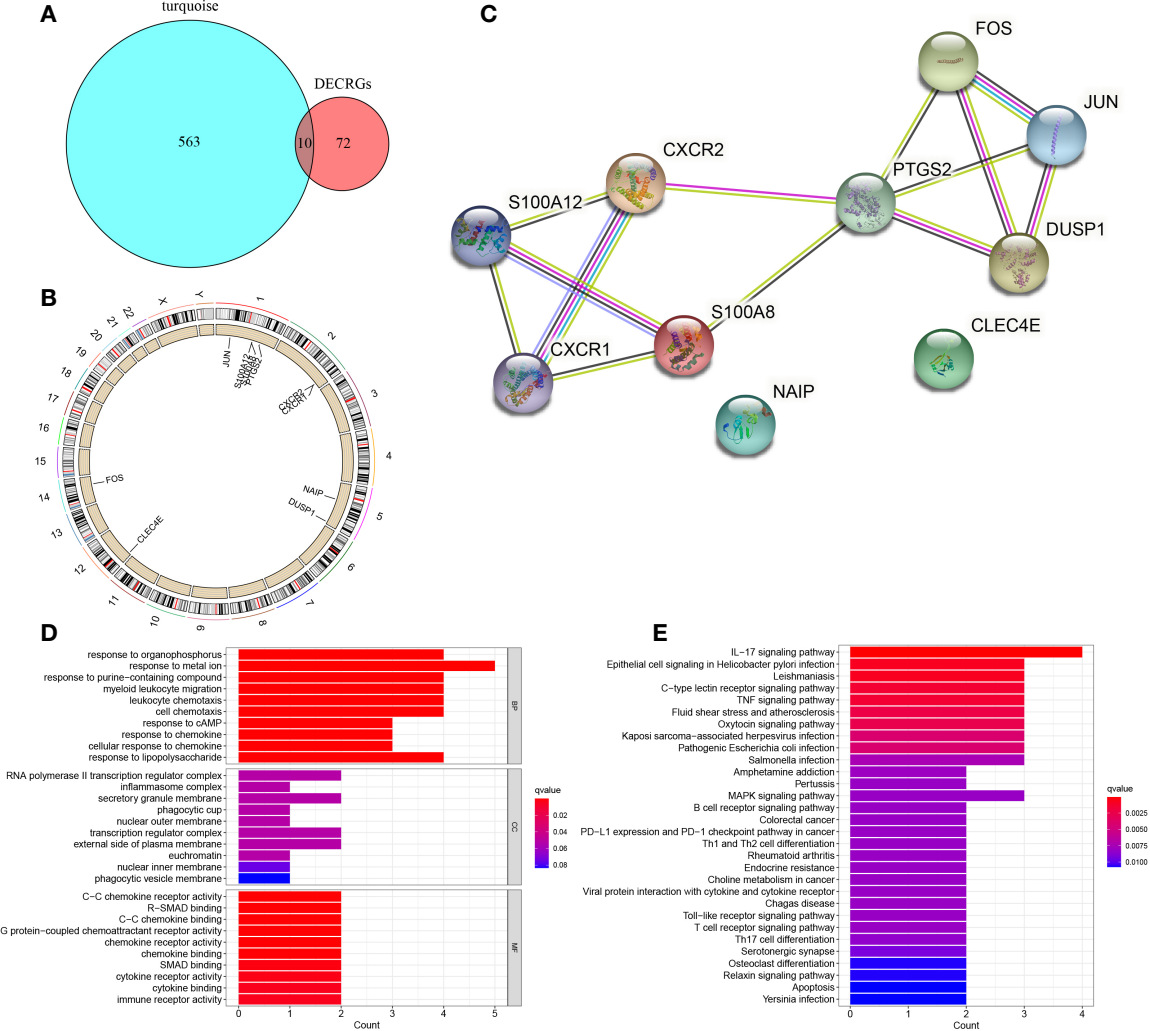
Identification of 10 hub CRGs. **(A)** The 10 hub CRGs were obtained by determining the intersection of DECRGs and genes in the turquoise module. **(B)** The specific location of each CRG on the chromosome. **(C)** PPI network of the 10 hub CRGs (10 nodes and 13 edges). **(D)** GO terms in the enrichment analysis of the 10 CRGs. **(E)** KEGG terms in the enrichment analysis of the 10 CRGs.

By taking the intersection of DECRGs and genes in the turquoise module, 10 hub CRGs were identified. These were *PTGS2*, Fos proto-oncogene, activator protein 1 (AP-1) transcription factor subunit (*FOS*), Jun proto-oncogene, AP-1 transcription factor subunit (*JUN*), S100 calcium binding protein A12 (*S100A12*), S100 calcium binding protein A8 (*S100A*8), C-X-C motif chemokine receptor 1 (*CXCR1*), C-X-C motif chemokine receptor 2 (*CXCR2*), dual specificity phosphatase 1 (*DUSP1*), C-type lectin domain family 4 member E (*CLEC4E*), and NLR family apoptosis inhibitory protein (*NAIP*) (**Figure 5A**). The location of each hub CRG on the chromosome is presented in **Figure 5B** (*PTGS2-*1q31.1*, JUN*-1p32.1*, S100A12*-1q21.3*, S100A8*-1q21.3*, FOS*-14q24.3*, CXCR1*-2q35*, CXCR2*-2q35*, DUSP1*-5q35.1, *CLEC4E*-12p13.31, and *NAIP-*5q13.2). The PPI network of the DECRG expression products in DN were constructed using the STRING database. The network included 13 edges and 10 nodes (**Figure 5C**).

GO enrichment analysis revealed that in terms of BP, the 10 hub genes were mainly enriched in response to metal ion, response to organophosphorus, response to purine−containing compound; in terms of CC, the hub genes were prominently involved in RNA polymerase II transcription regulator complex, secretory granule membrane, and transcription regulator complex; with regard to MF, the hub genes were engaged in C−C chemokine receptor activity, R−SMAD binding, and C−C chemokine binding (**Figure 5D**). KEGG pathway analysis revealed the involvement of these genes mainly in the IL−17 signaling pathway, epithelial cell signaling in *Helicobacter pylori* infection, Leishmaniasis, C−type lectin receptor signaling pathway, and tumor necrosis factor signaling pathway (**Figure 5E**).

### Determination of regulatory signatures for 10 hub CRGs

**Figure 6:**
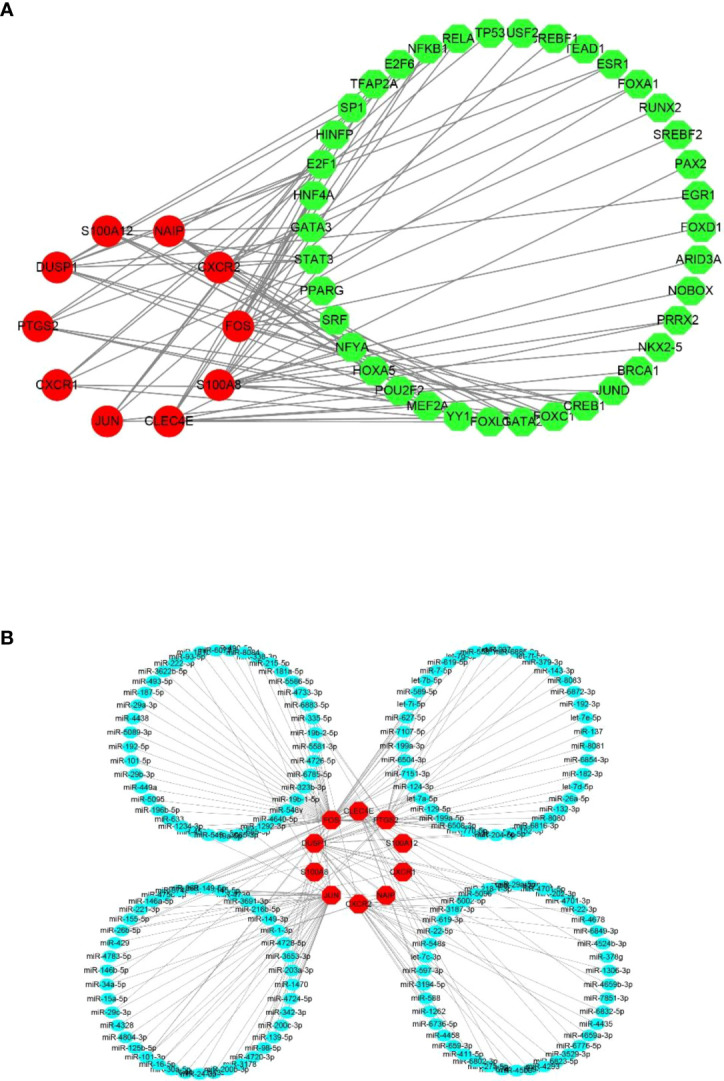
Potential regulatory molecules for the 10 hub CRGs. The red nodes depict the hub CRGs). **(A)** Ten hub CRGs and their interactions with potential transcription factors (TFs) based on the JASPAR database (49 nodes and 76 edges). **(B)** Ten hub CRGs and their interactions with potential miRNAs based on the miRTarBase database (175 nodes and 189 edges).

To gain insights into the CRG regulatory molecules and identify substantial changes at the transcriptional level, we employed publicly available bioinformatic database to reveal the potential TFs and miRNAs. Here, we present the results from the JASPAR and miRTarBase databases. The interaction of TFs and hub CRGs is depicted in **Figure 6A** and the interaction of potential miRNAs and hub CRGs is shown in **Figure 6B**. The TFs–CRGs and miRNAs–CRGs interaction network analysis revealed the regulatory activities of 39 TFs and 165 miRNAs with more than one CRGs, indicating strong interference between them. The specific regulatory molecules from JASPAR and miRTarBase for each hub CRG are summarized in **Supplementary Table 2**.

### Identification of hub regulatory molecules for two key CRGs

**Figure 7:**
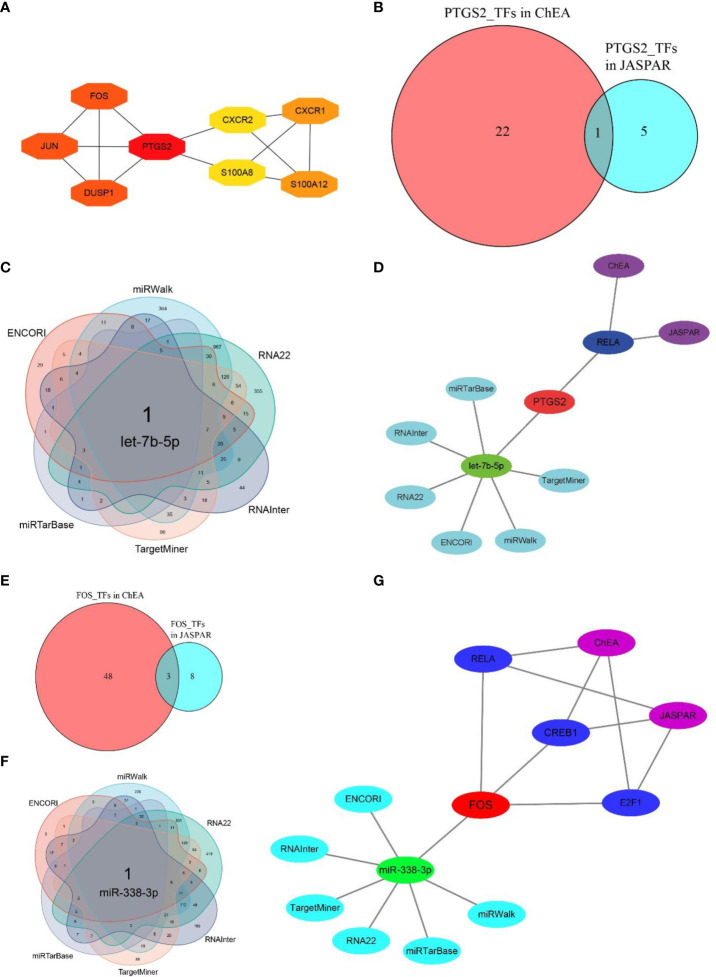
Determination of hub regulatory molecules for PTGS2 and FOS. **(A)** Top eight essential CRGs ranked by MCC scores (NAIP and CLEC4E are not shown because of the absence of interactions with other CRGs), and PTGS scores the highest among 10 hub CRGs. **(B)** One hub TF targeting PTGS2 was identified by taking the interaction of TFs from two databases (JASPAR and ChEA). **(C)** One hub miRNA targeting PTGS2 was identified by taking the intersection of miRNAs from six databases (miRWalk, RNA22, RNAInter, TargetMiner, miRTarBase, and ENCORI). **(D)** Hub TFs and miRNAs identified by multiple databases for PTGS2. **(E)** Three hub TFs targeting FOS were identified by taking the interaction of TFs of two databases. **(F)** One hub miRNA targeting FOS was identified by taking the intersection of miRNAs from six databases. **(G)** Hub TFs and miRNAs identified by multiple databases for FOS.

Based on MCC algorithm of cytohubba plugin in Cytoscape software, *PTGS2* scored the highest among 10 hub CRGs (**Figure 7A**). Aside from being the most significant DECRG according to the differential expression analysis (**Figure 3A**), *FOS* also ranked just behind *PTGS2* according to the MCC algorithm (**Figure 7A**). Hence, *PTGS2* and *FOS* were considered the two key CRGs. To find the hub regulatory molecules targeting these two key CRGs, the intersection of the TFs or miRNAs was determined from multiple databases.

By taking the intersection of the predicted results from both the JASPAR and ChEA databases, RELA proto-oncogene, NF-κB subunit (*RELA*), was screened as the hub TF interacting with *PTGS2*. After combining six databases, i.e., miRTarBase, ENCORI, RNAInter, TargetMiner, miRWalk, and RNA22, let-7b-5p was identified as the hub miRNA (**Figures 7B** **–D**). Similarly, cAMP responsive element binding protein (*CREB1*), E2F transcription factor (*E2F1*), and *RELA* were identified as the hub TFs for *FOS*, and miR-338-3p was screened as the hub miRNA (**Figures 7E** **–G**). The detailed regulatory molecules derived from each database for two key CRGs are summarized in **Supplementary Table 3**.

### Analysis of immune cell infiltration between normal and DN groups

**Figure 8:**
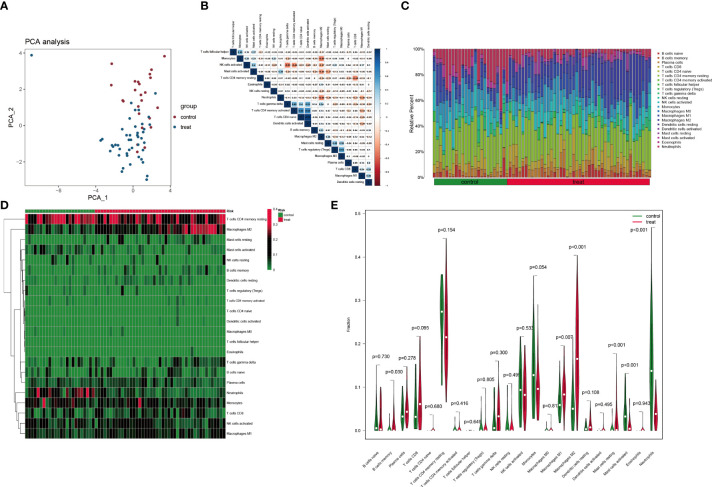
Immune cell infiltration analysis based on the CIBERSORT algorithm. **(A)** PCA performed on two groups. Red points indicate normal samples and green points indicate DN samples. **(B)** Correlation matrix of infiltration degree of immune cells in DN samples. **(C)** The abundance of different immune cells in each sample. **(D)** The infiltration degree of 22 immune cells in each sample. Red squares indicate higher immune infiltration expression and green squares indicate lower expression. **(E)** Violin plots of the differential analysis of different immune cells between two groups.

PCA indicated no overlap of two clusters, indicating a significant difference in immune cell infiltration between normal and DN samples (**Figure 8A**). Based on the CIBERSORT algorithm, the heatmap for correlation between immune cells is presented in **Figure 8B**. The most significant positive correlation was between activated dendritic cells and naïve CD4^+^ T cells (correlation coefficient = 0.93). A remarkable inverse correlation exists between T cells gamma delta and activated NK cells (correlation coefficient = -0.51). The relative proportion of various immune cells in each sample is shown in **Figure 8C**. Subsequently, we compared the expression level of different immune cells between two groups (**Figures 8D, E** **)**. In contrast to normal tissues, infiltration in DN tissue was statistically greater for memory B cells (*p* = 0.03), macrophages M1 (*p* = 0.007), macrophages M2 (*p*< 0.001), and resting mast cells (*p*< 0.001), while activated mast cells (*p*< 0.001) and neutrophils (*p*< 0.001) infiltrated statistically less in DN tissue.

### Correlation between *PTGS2* expression and immune cell infiltration

**Figure 9:**
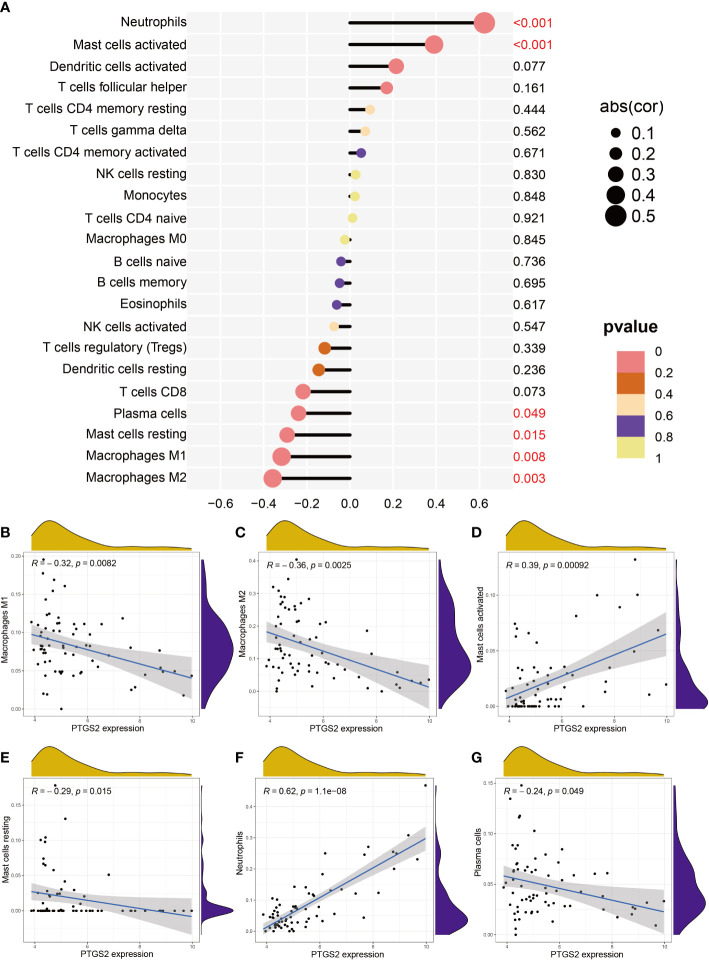
Correlation between expression PTGS2 expression and different immune cell infiltration degree in DN. **(A)** Lollipop plot. **(B–G)** Correlation between expression of PTGS2 and **(B)** M1 macrophages expression, **(C)** M2 macrophage expression, **(D)** activated mast cell expression, **(E)** resting mast cell expression, **(F)** neutrophil expression, and **(G)** plasma cell expression.

Next, we investigated the relationship between *PTGS2* and immunological characteristics. It was clear from the lollipop chart (**Figure 9A**) and scatter plot (**Figures 9B** **–G**) that *PTGS2* expression was significantly and positively associated with the level of infiltration of neutrophils (*R* = 0.62, *p*< 0.001) and activated mast cells (*R =* 0.39, *p<* 0.001) and negatively correlated with plasma cells (*R =* -0.24, *p* = 0.049), resting mast cells (*R =* -0.29, *p =* 0.015), macrophages M1 (*R =* -0.32, *p =* 0.0082), and macrophages M2 (*R = -*0.36, *p =* 0.0025).

## Discussion

In our study, the determination of the intersection of DECRGs and genes in the turquoise module (*R* = 0.77, *p<* 0.001) identified 10 intersection genes (*PTGS2*, *JUN*, *S100A12*, *S100A8*, *FOS*, *CXCR1*, *CXCR2*, *DUSP1*, *NAIP*, and *CLEC4E*). Subsequently, we analyzed the interaction of genes encoding TFs and miRNAs to discover transcriptional and post-transcriptional regulators of the intersection genes. TFs, including *STAT3 (* 43), *SP1 (* 44), *USF1 (* 45), *USF2 (* 46), *YY1 (* 47), *EGR1 (* 48), *FOXA1 (* 49), *E2F1 (* 50), *NRF1 (* 51), *SRF (* 52), *PPARG (* 53), *JUND (* 54), *TP53 (* 55), and *HNF4A (* 56), were identified as being associated with the development of DN. Further, some miRNAs involved in DN (e.g., miR-155-5p, miR-221-3p, miR-103-3P) (57–59), immune disorder (e.g., miR-4701-5p, miR-232b-3p, miR-338-3p) (60–62), and different cancers (e.g., miR-22-3p, miR-5096, miR-588, miR-29a-5p) (63–66) have been identified. TFs and miRNAs basically target main proteins to influence the progression of various diseases. Dou et al. (67) described the upregulation of miR-202-3p in type 1 gastric neuroendocrine neoplasms (g-NENs), which might initiate the pathogenesis of type 1 g-NENs by targeting *DUSP1.* Yao et al. found that miR-29c-3p targeted *FOS* to inhibit epithelial-mesenchymal transition and cell proliferation and contribute to apoptosis in transforming growth factor-beta 2 treated SRA01/04 cells (68). High miR-139-5p expression reportedly suppressed the c-Jun-vascular endothelial growth factor/platelet derived growth factor B pathway and reduced the migration of endothelial colony-forming cells isolated from patients with diabetes, tube formation, and proliferation. These events prevent diabetic vascular disease (69). miR-22-3p targets *CXCR2* and then contributes to injury induced by oxidized low-density lipoprotein (70). The majority of miRNAs are associated with cancer tissues and lead to various types of cancer in humans.

Based on the MCC algorithm, we identified *PTGS2* as the hub gene. This gene could be the key Cu-related biomarker or drug target and could be associated with pathophysiological mechanisms in DN. *PTGS*, also known as cyclooxygenase (*COX*), is the key gene responsible for prostaglandin biosynthesis from arachidonic acid, including prostaglandin E2, prostacyclin, prostaglandin 2 alpha, prostaglandin D2, and thromboxane. There are two *COX* isozymes: *COX-1* and *COX-2*. In the kidney, *COX-1* is constitutively expressed and is mainly present along the distal tubule. COX-1 mediates the synthesis of prostaglandin E2. The *COX-2* gene is inducible and shares significant homology with *COX-1*. *COX-2* is mainly expressed in both macula densa cells in the cortex and the thick limb of the ascending loop of Henle and medulla. Cortical COX-2 plays a pivotal role in renin release and subsequent pro-hypertensive effects, while COX-2 in the medullary region contributes to salt and water reabsorption (71). Accumulated evidence has associated the overexpression of *COX-2* with the progression of DN, with *COX-2* inhibition reducing hyperfiltration and proteinuria, abrogating Ang II-mediated reductions in GFR, and retarding progressive renal injury (72–75).

By determining the intersection of TFs or miRNAs from multiple databases, *RELA* (*p65*) and has-let-7b-5p were identified as the hub regulatory molecules. NF-κB, frequently under the form of *p50/p65 (RELA)* dimer, is a universal TF that can be post-transcriptionally activated by a number of stimuli, including infections, radiation, and oxidants, with pro-inflammatory effects (76, 77). A body of literature supports the view that the NF-κB signaling pathway serves as the link of Cu and *PTGS*. For example, Cu can activate and induce NF-κB-dependent genes, including *COX-2, NOS-II*, and *TNF-α*, through production of ROS (78). Liu et al. observed that the intragastrical provision of distilled water with dissolved copper sulfate (more than 4 mg/kg) to mice was able to activate the NF-κB signaling pathway and subsequently increase the expression of *COX-2* and prostaglandin E2, thereby inducing an inflammatory response in the liver (79). Similarly, Yang et al. observed that excessive Cu intake led to oxidative stress with activated NF-κB pathway and up-regulated *COX-2*, inducing inflammatory responses in immune organs of chickens (80). These findings indicate that anti-inflammatory therapy involving *COX-2* inhibition might be a promising therapeutic strategy for DN (74, 81–83). However, few investigators have linked this anti-inflammatory mechanism to the regulation of Cu homeostasis (84). let-7b-5p is down-regulated in different biological sources of patients with type 2 DM (85). Li et al. reported that let-7b-5p could translocate into mitochondria and lead to reduced ROS production by cardiomyocytes in db/db mice *via* the positive modulation of mitochondrial gene cytochrome b (86). In a prospective study incorporating 116 patients with type 1 DM, let-7b-5p was significantly associated with the increased risk of ESRD (Odds ratio = 2.38, *p =* 0.004) based on logistic regression analysis (87). However, no study has investigated the relationship of Cu, let-7b-5p, and *PTGS2*. Interestingly, the present PPI network analysis revealed a strong interaction of PTGS2 with CYP2B6. CYP2B6, accounting for approximately 3% to 6% of the total hepatic CYP content, is an important biotransformation enzyme for numerous clinical drugs and environmental toxins (88). A considerable interindividual variability in CYP2B6 protein expression has been observed, which is explained by genetic factors that include extensive genetic polymorphisms and non-genetic factors including some inhibitors and inducers (89).

In addition, we identified miR-338-3p as the hub miRNA for *FOS*, and *CREB1*, *E2F1*, and *RELA* were screened as the hub TFs. The *Fos* gene family is composed of four members: *FOS*, *FOSB*, *FOSL1*, and *FOSL2*. These genes encode leucine zipper proteins that dimerize with the *JUN* family proteins to form activator protein 1 (AP-1) TF (90). *AP-1* activation contributes to the accumulation of mesangial cell proliferation, extracellular matrix production, and subsequent progressive fibrosis in DN (91, 92). Expression changes of miR-338-3p occur long before the onset of diabetes. The overexpression of miR-338-3p is able to inhibit tumor necrosis factor-alpha (TNF-α)-mediated hepatic gluconeogenesis and rescue TNF-α-induced insulin resistance by targeting protein phosphatase 4 regulatory subunit 1 (93–95). E2F transcription factor 1 (E2F1) is crucial in the regulation of DNA synthesis and cell cycle. E2F1 promotes hepatic gluconeogenesis and hyperglycemia through the cyclin dependent kinase 4-Rb1-E2F1 pathway and downstream activation of phosphoenolpyruvate carboxykinase 1 (96). *CREB1* is a key TF in the basic leucine zipper class. The elevated phosphorylation level of CREB1 can promote the synthesis of fibronectin in the mesangial cells treated with high glucose (25 mmol/L) (97). Although some reports suggested that inhibition of CREB reduces blood glucose in the liver (4, 11, 12), another study reported that CREB is not necessary for the regulation of hepatic glucose metabolism (98). Therefore, the role of CREB in the regulation of hepatic gluconeogenesis is still controversial.

In the present study, immune cell infiltration analysis revealed significant differences in macrophages M1 and M2, resting mast cells, activated mast cells, and neutrophils between normal and DN samples. Macrophages are the most prevalent infiltrating immune cells in the kidney of DN patients. Despite the elimination of apoptotic cells or any other foreign pathogens by phagocytosis or T cells activation, macrophages are closely associated with a decline in renal function (99). Traditionally, macrophages are classified as classically activated M1 phenotype and alternatively activated M2 phenotype. M1 is the predominant phenotype at the site of diabetic kidney injury, which mediates the initiation phase of inflammation, tissue damage, and renal fibrosis through proinflammation cytokines that include TNF-α, interleukin (IL)-6, IL-10 and monocyte chemoattractant protein 1 (100, 101). M2 is involved in the wound healing process through production of anti-inflammatory cytokines, growth factor, and proangiogenic cytokines. Switching from the M1 to M2 phenotype may effectively reduce podocyte injury, albuminuria, and glomerulosclerosis and protect the kidney against DN (102–105). *PTGS2* stimulates a tissue-repair M2 phenotypic changes in macrophages (101). There is also a close interaction between mast cells and development of DN, and the vast majority of mast cells are located in the peritubular, perivascular, and periglomerular interstitial regions rather than in intraglomerular areas where inflammatory cells accumulate. Through release of some bioactive substances, such as tryptase, chymase, transforming growth factor-beta 1, renin, and TNF-α, into the tubular interstitium by degranulation, mast cells can lead to renal inflammation, fibrosis, and the progression of DN (106). There are very few studies on the role of neutrophils in DN. Some researchers suggested that a high blood neutrophil to lymphocyte ratio is a predictor of poor GFR in patients with diabetes (107, 108).

There are two limitations of our study. First, due to the small sample size and database limitations, we cannot externally validate our results. Second, there is a paucity of confirmatory experiments. Further fundamental and prospective studies would be beneficial.

## Conclusion

We identified 10 hub Cu-related genes (*PTGS2*, *FOS*, *DUSP1*, *JUN*, *S100A8*, *S100A12*, *NAIP*, *CLEC4E*, *CXCR1*, and *CXCR2*). The findings provide insight into the mechanisms of DN development at the transcriptome level. It is beneficial to examine and understand the interaction between hub Cu-related genes and potential regulatory molecules in DN, which may provide a novel theoretical foundation for the development of diagnostic biomarkers and copper-related therapy targets in DN. Further relevant molecular biological experiments are needed to confirm the function of the identified genes associated with DN.

## Data availability statement

The original contributions presented in the study are included in the article/**Supplementary Material**. Further inquiries can be directed to the corresponding author.

## Author contributions

JM: Investigation, Data Collection and Analysis, Writing-Original Draft and Editing; SS: Protocol Development, Writing-Editing; and XD: Supervision. All authors contributed to the article and approved the submitted version.

## Funding

This work was supported by Beijing Xinyue Foundation (2022IIT037) awarded to Sana SRGL.

## Conflict of interest

The authors declare that the research was conducted in the absence of any commercial or financial relationships that could be construed as a potential conflict of interest.

## Publisher’s note

All claims expressed in this article are solely those of the authors and do not necessarily represent those of their affiliated organizations, or those of the publisher, the editors and the reviewers. Any product that may be evaluated in this article, or claim that may be made by its manufacturer, is not guaranteed or endorsed by the publisher.
